# GE-Detection: Efficient Attention and Dropout for Low-Light Object Detection

**DOI:** 10.3390/s26123909

**Published:** 2026-06-19

**Authors:** Xiaochen Li, Hongtian Zhao

**Affiliations:** College of Mathematics and System Science, Xinjiang University, Huari Street, Urumqi 830017, China; lixiaochen@stu.xju.edu.cn

**Keywords:** object detection, global sub-sampled attention, efficient multi-scale attention, dropout regularization, low-light detection

## Abstract

Object detection in low-light scenes is difficult because weak illumination reduces local contrast, amplifies sensor noise, and makes small or occluded objects hard to localize. Existing enhancement-before-detection pipelines can improve visual brightness, but they are not always optimized for detection features, while transformer-style global reasoning is often too costly for lightweight detectors. To address this gap, we propose GE-Detection, a detector-side framework that integrates Global Sub-Sampled Attention (GSA), Efficient Multi-scale Attention (EMA), and dropout regularization into YOLO- and PicoDet-style architectures. GSA introduces lower-cost global context modeling through spatially reduced key-value tokens, EMA refines multi-scale fused features without aggressive channel compression, and dropout improves training-time regularization with no inference-time parameter overhead. Experiments on COCO, ExDark, BDD100K-Night, and NightOwls show that the method is most effective in low-light detection: on ExDark with YOLO11n, mAP50-95 improves from 34.39% to 36.74%, mAP50 from 56.24% to 59.27%, and Box (P) from 67.63% to 71.36%. The full YOLO11n variant uses 2.91M parameters and maintains 134.7 FPS on an RTX 2080 Ti under the tested setting. Cross-dataset and corruption experiments further indicate that the proposed modules improve localization under several nighttime domain shifts while retaining known limitations under severe noise and adverse weather. These results indicate that combining efficient global attention, multi-scale feature recalibration, and targeted regularization can improve low-light localization while keeping the detector practical for deployment.

## 1. Introduction

Object detection [1,2,3] is a key problem in computer vision and has progressed with deep learning. Current detectors are commonly built on two architectural paradigms: attention-based models, represented by Vision Transformers [4], and convolutional detectors, including the You Only Look Once (YOLO) family [5]. Attention mechanisms are well suited to modeling long-range dependencies, but their computational cost is high on edge devices, especially when dense prediction requires high-resolution feature maps. CNN-based detectors such as YOLO are more efficient and easier to deploy, but they cannot capture contextual relationships between distant objects well. This trade-off comes mainly from the tension between efficient computation and global context modeling. It is further affected by limited regularization, which can make detectors prone to overfitting when training data are scarce, imbalanced, or visually degraded. Reliable generalization in extremely dark environments therefore remains difficult across deployment settings.

Low-light object detection is not simply a standard detection task applied to darker images. Photon scarcity lowers signal-to-noise ratio, camera gain can amplify read noise, compression and demosaicing artifacts become more visible, and object boundaries may disappear into dark backgrounds. These factors are especially harmful for small objects, partially occluded objects, and crowded scenes because the detector must infer both category semantics and precise box boundaries from weak local evidence. Unlike image enhancement, detection also requires preserving discriminative features and geometric cues rather than only improving human-perceived brightness.

Many lightweight attention modules used in CNN-based detectors, including channel-attention and hybrid channel-spatial designs such as squeeze-and-excitation (SE), convolutional block attention module (CBAM), and coordinate attention (CA), rely on explicit channel compression to model inter-channel dependencies. Although this squeeze operation reduces computation, it maps high-dimensional responses to a narrow bottleneck and may remove fine-grained semantic cues that are important for detecting small, dim, or heavily occluded objects. This problem is particularly relevant to object detection, where class-specific patterns and localization cues need to be preserved across scales rather than compressed too early. If useful channels are suppressed during early feature recalibration, later aggregation stages can recover only part of the lost information, which may reduce robustness in low-light and cluttered scenes. Efficient Multi-scale Attention (EMA) offers a useful alternative because it models cross-spatial interactions and multi-scale dependencies without severe channel reduction. It can therefore preserve richer feature diversity while maintaining computational efficiency, making it suitable for practical detectors that must operate under limited computational budgets while retaining spatial sensitivity.

To address these issues, we propose an enhanced object detection model (GE-Detection) that integrates global sub-sampled attention (GSA), Efficient Multi-scale Attention (EMA), and dropout regularization. GSA reduces the cost of global reasoning by sub-sampling attention regions, allowing the detector to capture broad contextual information without incurring the full cost of dense self-attention within the attention module. EMA strengthens cross-spatial interaction and multi-scale context aggregation while avoiding the information loss often caused by channel compression. Dropout regularizes feature activations and reduces overfitting under difficult illumination and occlusion, while leaving the inference-time architecture essentially unchanged. We evaluate the proposed integration on COCO, ExDark, BDD100K-Night, and NightOwls, and further test cross-dataset transfer and corruption robustness. The results show improvements for several YOLO variants, with more consistent gains in localization precision on ExDark and more model-dependent AP changes on COCO. This contrast suggests that the method should be viewed primarily as a low-light-oriented enhancement, rather than as a modification that uniformly improves all detector scales.

The main research gap addressed in this paper is the lack of a lightweight detector-side design that jointly considers global context, multi-scale feature preservation, and regularization under low-light degradation. Enhancement-based methods can introduce visually pleasing images but may alter texture statistics used by detectors. Heavy transformer detectors can model global context but are often expensive for real-time deployment. Conventional attention blocks used in CNN detectors may compress channels too aggressively, which can remove weak low-light cues. GE-Detection is designed to occupy the middle ground: it adds global reasoning and multi-scale recalibration to existing detectors while keeping parameter and latency overhead moderate.

The theoretical motivation for combining GSA, EMA, and dropout is that each component addresses a different source of low-light detection error. GSA reduces the quadratic burden of global attention and helps use scene-level context when local evidence is weak. EMA preserves and recalibrates multi-scale spatial responses without severe channel bottlenecks, which is important for dim small objects and blurred boundaries. Dropout reduces co-adaptation during training and can improve generalization when low-light data are limited or visually heterogeneous. Their integration is therefore not a simple stacking of modules, but a combination of context modeling, feature preservation, and regularization.

The contributions of this work are summarized as follows:

- We propose a unified detector-side framework that combines GSA, EMA, and dropout regularization in YOLO- and PicoDet-style detectors for low-light object detection.
- We provide a mathematical analysis of the GSA spatial-reduction window and explain why stage-wise window sizes are more suitable than a single fixed reduction rule for multi-stage detection feature maps.
- We report component, grouping, branch, multi-seed, efficiency, cross-dataset, and low-light baseline comparisons to separate accuracy gains from parameter and runtime overhead.
- We evaluate reproducibility, dataset selection, failure cases, and robustness behavior under noise, blur, weather, compression, and camera variation.

### 1.1. Related Work

#### 1.1.1. Low-Light Enhancement and Low-Light Detection

Low-light vision methods can be broadly divided into image enhancement methods and detection-oriented methods. RetinexNet [6] decomposes low-light images into illumination and reflectance components, while Zero-DCE [7] learns curve-based enhancement without paired supervision. These methods can improve image visibility, but their training objectives are usually closer to perceptual enhancement than to bounding-box localization. Transformer-based enhancement systems such as Retinexformer [8] further improve restoration quality by combining Retinex modeling with long-range dependency learning. However, enhancement-first pipelines may amplify noise, change texture statistics, or produce artifacts that are not necessarily beneficial for detectors.

Recent low-light detection methods move closer to the detection objective. NLE-YOLO [9] and YOLO-AS [10] adapt YOLO-style detectors for dark environments, while NF-DETR [11] explores a transformer-based nighttime detector with frequency-domain enhancement. These studies show the value of task-aware adaptation under adverse illumination. Compared with these methods, our work focuses on modular integration into existing YOLO/PicoDet detectors, emphasizing efficient global context, multi-scale feature preservation, and training regularization rather than a fully new detector backbone.

#### 1.1.2. Attention Mechanisms in Object Detection

Attention-based models, including Vision Transformer (ViT) [4] and Detection Transformer (DETR) [12], have been widely studied in visual recognition and object detection because self-attention offers a direct way to model global context. ViT [4] captures long-range interactions among visual tokens, but its quadratic complexity limits scalability in dense prediction, especially for high-resolution inputs with many spatial tokens. DETR [12] formulates object detection as a set prediction problem through a transformer encoder–decoder architecture, reducing the need for hand-designed detection components, although its training convergence is often slow. Later architectures, such as the shifted-window design in Swin Transformer [13], lower the computational cost by restricting attention to local windows, but this locality can weaken global contextual consistency when distant objects or scene-level cues are important. Subsampling-based methods, such as token pruning in DynamicViT [14], pursue a different efficiency trade-off by identifying redundant visual tokens instead of processing all tokens uniformly. In this work, we use GSA as an implementation-level component rather than a new transformer backbone. It is introduced to prioritize informative regions while preserving image-level context at a lower attention cost. Related studies [15,16,17,18,19] also show that attention modules can improve YOLO-based detectors in practical deployment settings.

A critical issue in attention-based detection is that global context must be balanced against dense-prediction cost. Deformable DETR [20] reduces the burden of full attention by sampling a sparse set of relevant spatial locations, which is conceptually related to our use of spatial reduction in GSA. The difference is that GE-Detection does not replace the full detector with a transformer detector; instead, it inserts efficient attention modules into established one-stage detectors. This design keeps the method compatible with lightweight CNN-based deployment while still improving access to non-local context.

#### 1.1.3. Efficient Multi-Scale Attention

Ouyang et al. [21] proposed Efficient Multi-scale Attention (EMA) to address limitations of conventional channel-attention and hybrid-attention modules. Unlike SE-, CBAM-, and CA-based designs, which often use channel reduction to model inter-channel dependencies, EMA preserves richer feature information by splitting channels into groups and folding part of the channel dimension into the batch dimension. This reshaping keeps more channel-specific responses available during feature recalibration and reduces the information bottleneck introduced by aggressive compression, which is particularly relevant to dense prediction. In object detection, this property is useful because accurate localization depends on both spatial cues and semantic responses. EMA also uses parallel multi-scale branches to capture short- and long-range contextual cues, and its cross-spatial learning strategy combines branch outputs to model pixel-level pairwise interactions more effectively [21].

#### 1.1.4. Non-Attention Architectures

CNN-based detectors [22], including YOLO [5] and Fast R-CNN [23], remain central to object detection because their inductive biases support efficient local feature extraction. At the same time, hierarchical convolutions do not explicitly model long-range contextual relationships, which can limit performance in cluttered scenes where broader context is needed to disambiguate objects. Hybrid designs such as GCNet [24] add global context modules to CNNs, but they introduce extra architectural components and require careful efficiency–accuracy trade-offs. In contrast, this work uses attention as a lightweight complement to CNN backbones, aiming to improve spatial reasoning while preserving computational efficiency.

YOLO and PicoDet have also become important practical detector families. YOLO models [5] are widely used because they offer a strong balance between detection accuracy and inference speed, and many versions and application-specific extensions have been proposed [25,26,27,28,29,30,31,32]. Lightweight and task-oriented variants further show how one-stage detectors can be adapted to constrained deployment settings. YOLO-LITE [33,34] targets real-time detection on non-GPU devices, transformer-enhanced YOLO explores the integration of attention into YOLO-style pipelines [15], Tinier-YOLO improves real-time detection efficiency [35], and YOLOX advances anchor-free one-stage detection [36]. Fast YOLO [37] improves inference speed, while ALPR-YOLO [27] adapts YOLO to real-time automatic license plate recognition.

#### 1.1.5. Regularization for Robust Detection

Dropout, introduced by Srivastava et al. [38], is one of the most widely used regularization methods for reducing overfitting by randomly suppressing parts of the feature representation during training. In object detection, however, its use is usually more limited and often remains relatively simple. One reason is that detection poses task-specific challenges, including class imbalance, spatially correlated features, and large variation in object scale. These factors make naive regularization risky, since excessive feature suppression can remove localization cues needed to detect small or occluded objects. In our framework, dropout is applied to selected attention layers and feature pyramid features. This design provides regularization for both spatial and semantic representations under challenging detection conditions, while leaving the inference-time architecture unchanged.

## 2. Model and Settings

**Figure 1:**
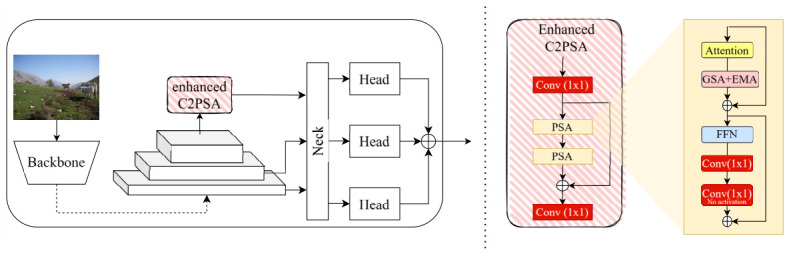
Architecture of the modified YOLO model.

**Figure 2:**
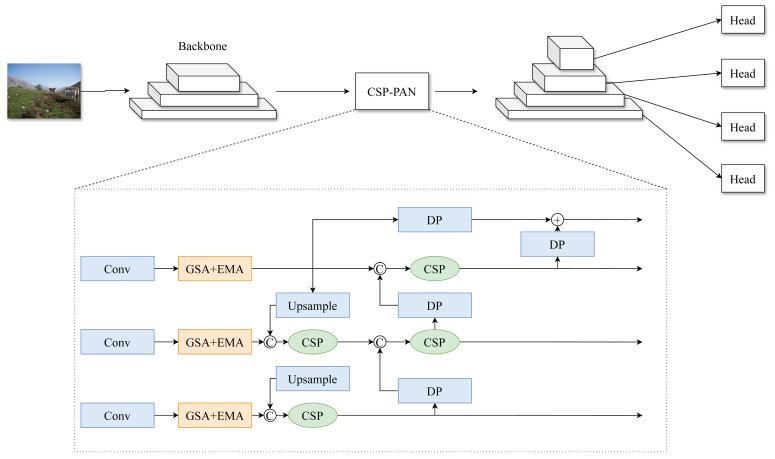
Architecture of the modified PicoDet model.

This section first describes the architectural modifications made to YOLO and PicoDet and then refers to the corresponding vector diagrams in Figure 1 and Figure 2. The figures are used to summarize where GSA, EMA, and dropout are inserted, while the following subsections provide the mathematical and implementation details of each module.

### 2.1. Overall Framework and Module Placement

GE-Detection is implemented as a modular modification rather than as a new detector family. For YOLO-style models, the attention refinement is inserted into the high-level C2PSA-related representation path, where semantic context is sufficiently rich and the feature resolution is lower than the raw input. For PicoDet-style models, the GSA + EMA blocks are placed around the feature-fusion path of the CSP-PAN neck, where features from different resolutions are merged before prediction heads. In both detector families, the goal is to improve contextual reasoning before final classification and box regression while preserving the original prediction head and loss design.

The three components play complementary roles. GSA provides low-cost global context by reducing the number of key-value tokens used in attention. EMA is applied after feature fusion to recalibrate multi-scale spatial responses without compressing channels into a severe bottleneck. Dropout is used only during training in selected attention and feature-refinement paths, so it regularizes feature learning without increasing inference-time parameters or FLOPs. This placement strategy is deliberately conservative: it avoids changing anchor/assignment rules, detection losses, non-maximum suppression, and the downstream evaluation protocol, which improves reproducibility and makes the effect of the inserted modules easier to isolate.

For an input image, the detector first extracts backbone features at multiple resolutions. The neck aggregates these features, after which EMA refines local and cross-spatial responses. GSA then introduces non-local information with stage-dependent spatial-reduction windows. The refined features are passed to the original detection heads for category prediction and bounding-box regression. The overall computational overhead is therefore mainly determined by the introduced GSA attention operations and the lightweight EMA convolutions, while dropout contributes no inference-time cost. Detailed quantitative results are reported in Section 3.

### 2.2. Global Sub-Sampled Attention

Following the spatial-attention design of Chu et al. [39], we use global sub-sampled attention (GSA) to reduce the module-level cost of global self-attention. The main idea is to summarize spatial regions before constructing attention keys and values, allowing the model to access global context with fewer token interactions. Chu et al. show that this form of spatial attention is effective across several vision tasks, including image classification and dense prediction tasks such as object detection and segmentation. We briefly review its formulation before describing how it is adapted to our detector.

A common way to enable information exchange across local groups is to add global self-attention layers after local attention blocks. For high-resolution feature maps, however, this quickly becomes expensive, with complexity $O(H^{2}W^{2}d)$, since all spatial positions interact densely. GSA reduces this cost by summarizing each $k_{1}\times k_{2}$ spatial region through a sub-sampling operation and using the reduced feature map to construct the attention keys and values. The query positions remain aligned with the original feature map, while the number of key-value tokens used in attention is reduced. Let the original sequence length be N=HW, and let the reduced sequence length after spatial reduction be $N^{\prime }=\frac{HW}{k_{1}k_{2}}$. The attention cost is reduced from $O(N^{2}d)$ to

(1) $$O\left(NN^{\prime }d\right)=O\;\left(\frac{H^{2}W^{2}d}{k_{1}k_{2}}\right).$$

Including the cost of spatial reduction, the total computational overhead is

(2) $$O\;\left(\frac{H^{2}W^{2}d}{k_{1}k_{2}}+k_{1}k_{2}HWd\right).$$

By the arithmetic–geometric mean inequality, the corresponding expression satisfies

(3) $$\frac{H^{2}W^{2}d}{k_{1}k_{2}}+k_{1}k_{2}HWd\geq 2HWd\sqrt{HW}.$$

Under a continuous relaxation, this lower bound is attained when $k_{1}k_{2}=\sqrt{HW}$. If the sub-sampling windows are assumed to be square, with $k_{1}=k_{2}$, this gives

(4) $$k_{1}=k_{2}={\left(HW\right)}^{1/4}.$$

For a standard image-classification input with H=W=224, the corresponding value is approximately $k_{1}=k_{2}\approx 15$. In detection, however, the feature-map resolution changes across stages, so the square and uniform-map derivation is used only as a resolution-aware guideline rather than as a single fixed architectural rule. For Stage 1, where the feature map has resolution 56×56, the square-window optimum is $k_{1}=k_{2}\approx \sqrt{56}\approx 7.48$, so a nearby integer such as 7 or 8 is appropriate in practice. We use $k_{1}=k_{2}=7$ for the highest-resolution stage, where the token count is largest. To avoid degrading the key-value representation in later stages, where the feature maps are already compact, we set the summarization window sizes of the last three stages to 4, 2, and 1, respectively. The fixed-window comparison is evaluated experimentally in Section 3.

The GSA module can be written more explicitly as a sequence of tokenization, spatial reduction, reduced key-value construction, global attention, and residual fusion. Let the input feature map at a detector stage be $F^{l}\in R^{B\times C\times H\times W}$. After flattening the spatial dimensions and projecting the channel dimension to *d*, we obtain

(5) $$x=\mathrm{Flatten}\left(F^{l}\right)W_{x},\;x\in R^{B\times N\times d},\;N=HW.$$

The query branch preserves all spatial positions, while the key and value branches are computed from a reduced token sequence. For the *j*-th spatial window $\Omega_{j}$ with size $k_{1}\times k_{2}$, the reduction operation is

(6) $$x_{\mathrm{sr},j}=\mathrm{LN}\;\left(\frac{1}{|\Omega_{j}|}\sum_{i\in \Omega_{j}}x_{i}\right),\;\left|\Omega_{j}\right|=k_{1}k_{2},$$

where LN(·) denotes layer normalization. This operation may be implemented by average pooling or an equivalent strided spatial-reduction layer. It keeps one representative token for each local region, so the number of key-value tokens becomes $N^{\prime }=HW/\left(k_{1}k_{2}\right)$. The query, key, and value tensors are then

(7) $$\begin{array}{cc} Q & =xW_{Q},\;Q\in R^{B\times N\times d}, \end{array}$$

(8) $$\begin{array}{cc} K & =x_{\mathrm{sr}}W_{K},\;K\in R^{B\times N^{\prime }\times d}, \end{array}$$

(9) $$\begin{array}{cc} V & =x_{\mathrm{sr}}W_{V},\;V\in R^{B\times N^{\prime }\times d}. \end{array}$$

For a single attention head with dimension $d_{h}$, the reduced global attention map and output are

(10) $$\begin{array}{cc} A & =\mathrm{softmax}\;\left(\frac{QK^{⊤}}{\sqrt{d_{h}}}\right),\;A\in R^{B\times N\times N^{\prime }}, \end{array}$$

(11) $$\begin{array}{cc} O & =\mathrm{Dropout}(A)V.\; \end{array}$$

For multi-head attention, the head outputs are concatenated and projected back to the detector feature dimension:

(12) $$\mathrm{GSA}\left(x\right)=\mathrm{Dropout}\;\left(\mathrm{Linear}\left(\mathrm{Concat}(O_{1},\ldots ,O_{h})\right)\right).$$

Finally, the GSA response is fused with the original stage representation by a residual connection,

(13) $${\hat{z}}^{l+1}=z^{l}+\mathrm{GSA}\;\left(\mathrm{LayerNorm}(z^{l})\right).$$

This formulation shows that GSA does not remove the original spatial query positions. Each pixel-level query can still attend to a global set of region-level key-value tokens, but the expensive N×N dense attention matrix is replaced by an $N\times N^{\prime }$ matrix. Therefore, the module keeps long-range contextual access while reducing the dominant attention cost. In low-light detection, this is useful because weak local boundaries often need support from scene-level cues, such as the relationship between a dark object, nearby structures, and the surrounding illumination pattern.

The attention matrix in 10 also provides a diagnostic interpretation of the module. A reduced token with a high column response

(14) $$s_{j}=\frac{1}{N}\sum_{i=1}^{N}A_{ij}$$

indicates that many spatial queries use information from the corresponding region-level key-value token. Conversely, dispersed responses indicate that the detector relies on broader contextual evidence. We use this response pattern qualitatively to interpret whether GSA focuses on informative object regions and surrounding context in dark scenes. Larger spatial-reduction windows reduce computation but may merge weak small-object evidence with background texture, whereas smaller windows preserve local detail at higher cost. This trade-off motivates the stage-wise setting: stronger reduction is used in high-resolution stages, and weaker reduction is used after feature maps become compact.

### 2.3. Efficient Multi-Scale Refinement

To further improve the detector’s multi-scale representation, we insert an Efficient Multi-scale Attention (EMA) module [21] after each neck fusion layer. This placement recalibrates fused features before they are passed to the prediction pathway at each scale, where semantic information and localization cues need to be balanced. Let the input feature tensor of the EMA block be $X\in R^{B\times C\times H\times W}$, and let the refined output be $Y\in R^{B\times C\times H\times W}$. We split the channel dimension of *X* into *g* groups, each containing C/g channels. The grouped tensor is then reshaped as

(15) $$\tilde{X}\in R^{(B\cdot g)\times (C/g)\times H\times W},$$

which allows each group to learn relatively independent semantic patterns while avoiding the information loss caused by aggressive channel reduction. The grouping factor *g* controls the granularity of feature specialization and cross-channel interaction.

Given $\tilde{X}$, EMA uses two parallel branches to extract complementary contextual cues. The first branch, denoted by $B_{1}\left(\cdot \right)$, applies a 1×1 convolution for lightweight feature interaction. The second branch, denoted by $B_{2}\left(\cdot \right)$, applies a 3×3 convolution to capture broader spatial dependencies. The two branches therefore emphasize fine-grained interaction and wider spatial context, respectively, which is useful for detection under scale variation and cluttered backgrounds.

After branch-wise feature extraction, EMA uses cross-spatial interaction to fuse the two responses. The outputs of the two branches are globally encoded and transformed into compatible matrix forms, and matrix multiplication is used to generate cross-spatial correlation maps between $B_{1}\left(\tilde{X}\right)$ and $B_{2}\left(\tilde{X}\right)$. These maps describe how complementary branch responses reinforce each other across spatial positions, rather than treating the branches as independent feature transformations. The resulting attention response is normalized by a sigmoid activation and used to reweight the grouped features. The refined features are then reshaped back to the original tensor size and fused with the input through a residual connection, which preserves the original detector representation while introducing attention-based recalibration. In our implementation, this refinement is written abstractly as

(16) $$Y=X+X⊙\sigma \;\left(\mathrm{Cross}\;\left(B_{1}\left(\tilde{X}\right),B_{2}\left(\tilde{X}\right)\right)\right),$$

where ⊙ denotes element-wise multiplication, σ(·) denotes the sigmoid function, and Cross(·) denotes cross-spatial interaction followed by reshaping to match the dimension of *X*. EMA preserves channel information, aggregates multi-scale spatial cues, and strengthens pixel-level contextual interaction with modest computational overhead, making it suitable for detector necks where efficiency and localization sensitivity must be considered together. The computational cost of EMA is dominated by the lightweight 1×1 and 3×3 branch convolutions applied to grouped features. Because the grouping operation reshapes channels rather than compressing them through a narrow bottleneck, EMA can preserve weak object cues that may otherwise be lost in low-light scenes. This is important for detection because localization errors often arise from subtle boundary and texture information rather than from class semantics alone.

## 3. Experiments

This section describes the experimental setup and evaluation protocol used to assess the proposed method. Although Figure 1 and Figure 2 give a high-level view of the modified architectures, we focus here on the training configuration and quantitative metrics used to compare detection accuracy and deployment cost. We then report results under complementary evaluation criteria, including standard detection accuracy, low-light detection performance, and parameter cost. These experiments are designed to evaluate the practical effect of the integrated framework in real-world and deployment-oriented settings.

### 3.1. Experimental Setup

The experimental setup covers model modification, dataset preparation [40,41], training hyperparameters, and evaluation criteria. COCO is used as a standard large-scale benchmark for general object detection, while ExDark is used to evaluate detection under low-light illumination. Large-scale training on COCO was conducted on a Tesla V100-PCIE-16GB GPU. The batch size was set to 64 for YOLO11n and 32 for the larger variants to fit GPU memory. Training on ExDark was performed on an RTX 2080 Ti 10GB GPU, with batch sizes of 16 for YOLO11n/s/m and 8 for the remaining models. For all settings using dropout, the dropout rate was fixed at 10% to reduce overfitting under the same training protocol. We used stochastic gradient descent (SGD) for optimization, with an initial learning rate of 0.01 and a momentum of 0.9. Unless otherwise specified, all models were trained for 300 epochs to keep the training budget consistent across model families. The reported values correspond to this fixed protocol rather than multi-seed averages.

To evaluate dataset coverage and domain generalization, the experimental protocol includes COCO, ExDark, BDD100K-Night, and NightOwls. Table 1 summarizes the evaluated datasets and their roles, while Table 2 reports the corresponding cross-dataset transfer results under matched metric definitions.

Table 3 compares baseline YOLO/PicoDet variants and their integrated GSA + EMA + Dropout counterparts on COCO and ExDark.

COCO and ExDark were selected for complementary reasons. COCO is a widely used general benchmark with diverse object categories and normal illumination, making it useful for checking whether the proposed modules damage standard detection behavior. ExDark is explicitly designed for object detection in low-light images and therefore directly evaluates the target scenario of this work. This pairing allows us to distinguish general detection behavior from low-light-specific gains.

**Figure 3:**
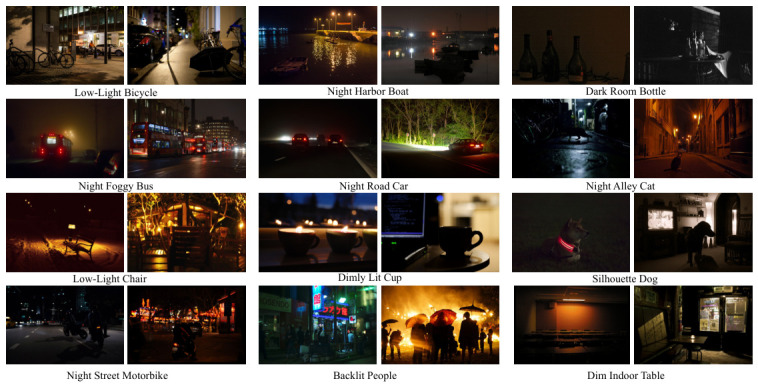
Representative images from the ExDark dataset. The examples show typical low-light degradations considered in this work, including weak illumination, low object-background contrast, partial occlusion, and visually cluttered backgrounds.

Figure 3 illustrates why ExDark is used as the main low-light benchmark. In these scenes, local edge and texture evidence is often weak, so a detector must combine local appearance with broader contextual information. This motivates using GSA to introduce global region-level context and EMA to refine multi-scale spatial responses without applying image-enhancement preprocessing.

**Figure 4:**
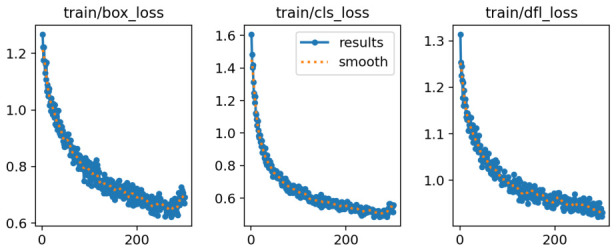
Validation losses of YOLO11n on ExDark.

**Figure 5:**
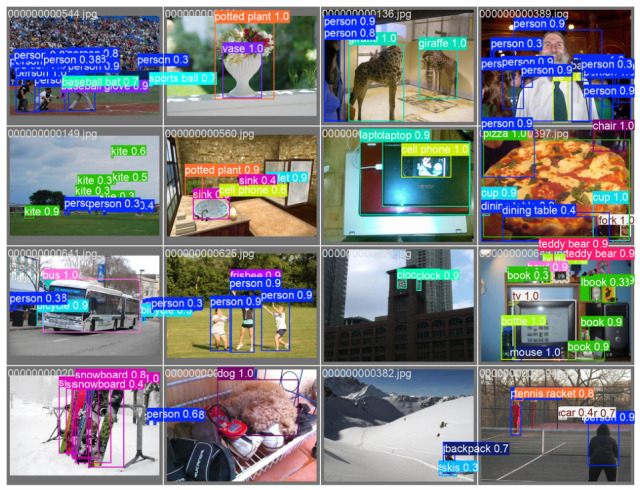
Visualization of YOLO11n inference on the COCO validation set. Colored bounding boxes indicate predicted object categories.

The training loss is shown in Figure 4, and the YOLO11n inference visualization is shown in Figure 5.

For reproducibility, all baseline and modified models are trained under matched settings within each detector family. YOLO models use an input size of 640×640, while PicoDet models use 416×416. Images are resized to the detector-specific input resolution before training and evaluation; no extra image-enhancement preprocessing is applied to the proposed method. Evaluation uses mAP50-95, mAP50, and bounding-box precision, with the same validation split and inference resolution used for each matched comparison. The only architectural changes are the insertion of GSA, EMA, and dropout at the positions described in Section 2, while the original prediction heads and detection losses are preserved. The implementation is publicly available; see the Data Availability statement for the repository link.

The cross-dataset experiments introduce both illumination shift and label-distribution shift, so class mapping is kept consistent before evaluation and the same mAP50-95, mAP50, and Box (P) metrics are used for each transfer setting. As shown in Table 2, GE-Detection improves over the corresponding baseline in all evaluated transfer directions, with gains of 2.0–2.3 percentage points in mAP50-95. These results support the external validity of the proposed modules, while also showing that cross-dataset performance remains lower than in-domain ExDark evaluation because nighttime driving and pedestrian datasets contain different camera viewpoints, exposure distributions, and object priors.

The qualitative analysis links the representative YOLO11n prediction visualization with later FP/FN auditing and robustness analysis. Side-by-side baseline/ours inspection is used to identify successful detections and remaining missed objects, while the false-positive and false-negative groups are quantified in the failure-case analysis below. GSA response inspection is used to check whether non-local context emphasizes informative dark-scene regions, and EMA response inspection is used to assess whether multi-scale spatial recalibration strengthens object-related features. These visual examples therefore support the explanatory discussion without changing the reported numerical protocol.

The proposed integration improves bounding-box precision in many cases, with the most evident gains appearing in several YOLO variants on ExDark. The improvements are not uniform across metrics or model families, which is an important distinction: stronger precision under low-light conditions suggests better localization reliability, whereas small or negative AP changes indicate that the integration does not benefit every operating regime equally. On ExDark, YOLO11n improves from 34.39% to 36.74% in mAP50-95 and from 67.63% to 71.36% in Box (P). For YOLO11l, Box (P) increases from 73.87% to 77.54%, while mAP50-95 changes only slightly, from 40.58% to 40.67%. These gains are obtained with a modest increase in model size; for example, YOLO11l grows from 25.43 M to 25.96 M parameters.

**Figure 6:**
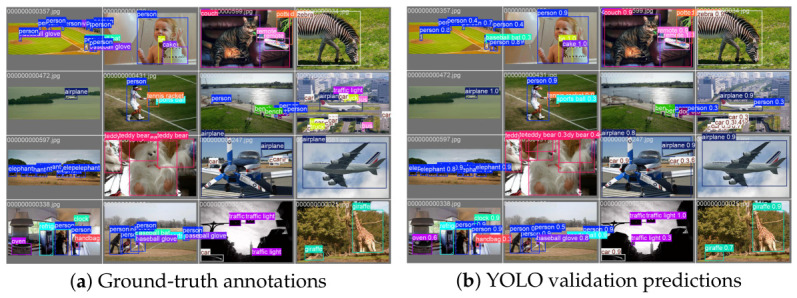
Qualitative validation visualization from the YOLO pipeline. The montage illustrates localization, confidence ranking, dense-object handling, and small-object sensitivity; the quantitative conclusions remain based on the benchmark and ablation tables.

**Figure 7:**
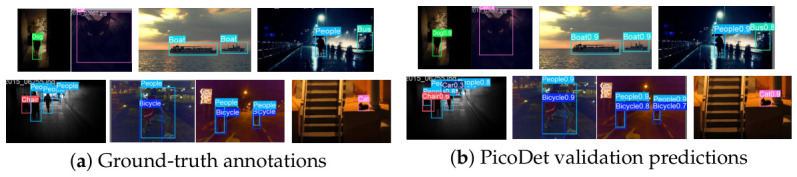
Qualitative visualization of PicoDet on the ExDark validation set. The comparison between ground-truth annotations and validation predictions illustrates detector behavior under weak illumination, glare, reflective water surfaces, low-contrast objects, and small distant targets.

The qualitative examples in Figure 5, Figure 6 and Figure 7 provide representative detector-output visualizations across YOLO and PicoDet settings. The label/prediction comparison in Figure 6 shows that the YOLO pipeline can localize many salient objects with high confidence, while crowded scenes, distant instances, and visually ambiguous regions still produce low-confidence boxes or missed detections. The PicoDet visualization in Figure 7 further highlights the difficulty of ExDark scenes: people, boats, bicycles, and cats remain detectable in many low-light examples, but confidence varies when objects are small, partially occluded, close to strong light sources, or embedded in dark backgrounds. These observations are consistent with the quantitative finding that the proposed modules improve localization precision more clearly than they uniformly improve all AP metrics.

The PicoDet variants show smaller changes across both datasets, again with only slight parameter growth. On COCO, YOLO11l reaches 53.65% mAP50-95, compared with 53.45% for the baseline, while the smaller YOLO variants do not consistently improve in mAP50-95. These results suggest that combining global context modeling, multi-scale refinement, and regularization is most useful for improving localization precision in low-light detection, but the magnitude of the gain depends on the model scale, backbone, and evaluation metric. All models are evaluated at their native input resolutions, namely 640×640 for YOLO and 416×416 for PicoDet.

The non-uniform gains are consistent with the different capacity and feature-fusion behavior of the tested detectors. Smaller models have limited representational capacity, so introduced attention modules may compete with the baseline feature extractor for optimization capacity. Larger YOLO variants already encode stronger semantic features, so GSA and EMA can mainly improve localization precision by refining context and multi-scale cues. PicoDet models are more compact and use a different lightweight detection design; therefore, the same inserted modules produce smaller changes. This indicates that GE-Detection is not a universal plug-in that guarantees AP improvement for every architecture, but a low-light-oriented refinement whose benefit depends on the balance between detector capacity, feature resolution, and degradation severity.

The stronger improvements in Box (P) than in all AP metrics suggest that the proposed modules mainly improve localization confidence and reduce false positive detections in dark scenes. However, AP also depends on recall, ranking quality, and performance across IoU thresholds. The method can therefore improve precision while producing smaller or even mixed changes in mAP50-95, especially when objects are extremely small, heavily occluded, or ambiguous under low contrast.

#### 3.1.1. Component-Wise Ablation of Attention and Regularization Modules

To assess whether the proposed modules improve the original detector beyond its baseline design, we compare GSA, EMA, dropout, and their combinations on ExDark under the same YOLO11n training protocol. The modules are inserted individually or jointly at their designated network positions, allowing us to examine their separate and combined effects under a controlled setting. This setup separates the roles of global context modeling, multi-scale feature recalibration, and training regularization before evaluating their joint behavior.

Table 4 reports the component-wise ablation results of GSA, EMA, and dropout on ExDark using YOLO11n. Because all variants follow the same training protocol and input resolution, the observed differences mainly reflect the effects of the inserted modules rather than changes in the optimization budget. Each individual component improves over the baseline, suggesting that global context modeling, multi-scale feature recalibration, and regularization all contribute to detection performance. Among the single-component variants, EMA gives the largest gain, increasing mAP50-95 from 34.39% to 35.92% and Box (P) from 67.63% to 70.08%. This result indicates that multi-scale spatial recalibration is particularly helpful on ExDark, where weak illumination and cluttered backgrounds make reliable localization more difficult. GSA also improves all three metrics, showing that global sub-sampled attention helps the detector capture longer-range spatial dependencies under low-light conditions. Dropout brings a smaller but consistent improvement, which suggests that training-time regularization can improve generalization without changing the feature extraction structure.

The combined variants show that the modules are complementary, although their effects are not strictly additive across all metrics. The combination of GSA and EMA achieves the best mAP50-95 and mAP50 in this ablation, reaching 36.31% and 58.82%, respectively. This improvement over either module alone suggests that global context modeling and local multi-scale refinement address different aspects of feature representation. Adding dropout to EMA also remains beneficial, with 36.18% mAP50-95 and 58.64% mAP50. When all three modules are used together, the model obtains the highest Box (P), increasing precision to 71.36%, although its AP scores are lower than those of the GSA + EMA variant. This result suggests that dropout in the full configuration favors localization precision more than overall AP in this setting. Overall, the ablation indicates that efficient global attention, multi-scale spatial interaction, and targeted regularization provide complementary benefits, but their best configuration depends on the evaluation metric being emphasized.

To evaluate the effect of dropout hyperparameters, we report a rate-and-placement ablation in Table 5. The comparison includes no-dropout references, multiple dropout rates at the proposed insertion points, and placement-specific variants in the backbone, neck/FPN, and detection head.

The dropout sweep shows that a rate of 0.10 at the proposed attention and feature-fusion locations gives the best overall result, reaching 36.74% mAP50-95, 59.27% mAP50, and 71.36% Box (P). Lower dropout (0.05) remains beneficial but is slightly weaker, while stronger dropout (0.15 and 0.20) gradually reduces AP, suggesting that excessive feature suppression can remove useful localization cues. The placement study further shows that neck/FPN dropout is more effective than backbone-only or detection-head-only dropout, which supports applying regularization near the multi-scale feature fusion path.

To further support the stage-wise GSA window heuristic derived in Equations (1)–(4), Table 6 compares the adopted resolution-aware setting (7,4,2,1) with fixed window sizes across all stages under the same YOLO11n ExDark protocol.

The window-size comparison shows that fixed and stage-wise settings are close in this YOLO11n ExDark setting. The fixed (1,1,1,1) setting obtains the highest AP but uses dense key-value interactions, whereas the adopted stage-wise (7,4,2,1) setting maintains competitive accuracy while following the intended efficiency-oriented design. This result indicates that the stage-wise rule is a practical trade-off rather than an absolute optimum for every metric.

#### 3.1.2. Ablation Study on EMA

To evaluate the standalone contribution of EMA, we conduct controlled ablation experiments on ExDark with YOLO11n as the base detector. These experiments isolate the internal design choices of EMA rather than compare different detector families. We examine the overall effect of adding EMA in Table 7, the influence of the channel grouping factor *g* in Table 8, and the role of the parallel branches and cross-spatial interaction in Table 9.

The ablation results show that EMA is effective in the YOLO11n setting on ExDark. Adding EMA increases mAP50-95 from 34.39% to 35.92% and Box (P) from 67.63% to 70.08%, indicating that the module provides useful feature refinement under this configuration. Since the detector, dataset, and training protocol are fixed, the gain can be attributed more directly to the feature recalibration introduced by EMA. The grouping study further shows that *g* affects the balance between feature diversity and interaction strength. Among the tested settings, g=16 gives the best observed performance, suggesting that overly coarse grouping may weaken feature specialization, while overly fine grouping may limit cross-channel aggregation. The component analysis also shows that neither the 1×1 branch nor the 3×3 branch alone is sufficient for the best detection performance. The full EMA variant performs best in this ablation, which suggests that lightweight feature interaction, broader spatial modeling, and cross-spatial fusion work together to improve perception in low-light and cluttered scenes under the tested detection setting.

### 3.2. Multi-Seed Stability Analysis

Because several AP differences are small, we also report the mean and standard deviation over three random seeds for representative configurations. For ExDark, we use YOLO11n to match the base model used in the component-wise ablation study. For COCO, we use YOLO11l because it shows the clearest gain among the YOLO variants in the main benchmark. This experiment is intended to separate stable trends from marginal variation caused by training randomness, rather than relying only on single-run comparisons. This analysis is especially relevant because the proposed integration yields larger gains on ExDark, while its effects on COCO are more moderate and metric-dependent.

As shown in Table 10, the full configuration improves performance consistently across three random seeds in the representative settings.

On ExDark with YOLO11n, it increases mAP50-95 from 34.42±0.17 to 36.69±0.15 and Box (P) from 67.69±0.24 to 71.28±0.22. These mean improvements are well above the observed run-to-run variation, suggesting that the gains on ExDark are unlikely to be explained by a favorable random seed alone. On COCO with YOLO11l, the changes are smaller: mAP50-95 and mAP50 improve slightly, while the gain in Box (P) is more visible. These results support the interpretation that the combination of GSA, EMA, and dropout provides more stable benefits for low-light detection, whereas its effect on general object detection is smaller and more dependent on the evaluation metric.

### 3.3. Efficiency and Deployment Cost

To support the deployment-oriented evaluation, we report computational cost and runtime behavior under a fixed inference setting. This analysis separates parameter overhead from practical inference cost, which is important for resource-constrained deployment because a small increase in parameters does not necessarily lead to a comparable change in latency or memory usage. All models are evaluated on an RTX 2080 Ti GPU with an input size of 640×640 and a batch size of 1. FPS and latency are measured in inference mode after warm-up, and memory refers to peak GPU memory usage during inference.

As shown in Table 11, the proposed modules introduce moderate computational overhead. Compared with the baseline, EMA increases the parameter count from 2.62 M to 2.74 M and the FLOPs from 6.50 G to 6.68 G, while maintaining an inference speed of 146.3 FPS. This result indicates that EMA improves feature representation at a small deployment cost. GSA introduces a larger overhead because it models long-range spatial dependencies, increasing FLOPs to 6.92 G and reducing FPS from 152.6 to 139.8. Even with this cost, the model retains real-time inference speed under the tested setting, suggesting that global context can be introduced without making inference impractical.

Dropout introduces no extra parameters or FLOPs during inference because it is disabled in evaluation mode. Its runtime cost is therefore almost identical to that of the baseline. The full model with GSA, EMA, and dropout has 2.91 M parameters and 7.10 G FLOPs, with 134.7 FPS and 7.42 ms latency. Although this configuration is slower than the baseline, the overhead remains limited relative to the accuracy gains observed in the ablation study. These results suggest that the proposed framework improves detection performance with a moderate deployment cost under realistic single-image inference settings, especially in low-light detection, where the accuracy gain is more pronounced.

The overhead analysis also clarifies which component is responsible for most of the deployment cost. EMA adds only 0.12 M parameters and 0.18 G FLOPs in the YOLO11n setting, whereas GSA adds a larger attention-related cost because it introduces long-range token interaction. The full model reduces FPS from 152.6 to 134.7, a relative decrease of approximately 11.7%, while improving ExDark mAP50-95 by 2.35 percentage points and Box (P) by 3.73 percentage points. This trade-off suggests that the full configuration is most appropriate when low-light localization reliability is more important than maximum throughput; for stricter real-time settings, EMA-only or GSA-window-adjusted variants may be preferable.

### 3.4. Comparison with Low-Light Detection Baselines

To clarify the scope of comparison, we include low-light-oriented baselines, covering enhancement-before-detection pipelines and recent detector variants designed for adverse illumination. This comparison examines whether the proposed integration is competitive with relevant low-light detection strategies under matched experimental conditions, rather than only with unmodified detector backbones. It also separates cascaded image enhancement from detection-oriented adaptation, since visual enhancement quality and detection accuracy are not necessarily optimized by the same objective.

The comparison also discusses Retinexformer [8], a transformer-based low-light enhancement method, and NF-DETR [11], a frequency-domain detection transformer for nighttime object detection. Retinexformer represents an enhancement-first transformer pipeline: it can improve scene visibility before detection, but detection accuracy may still be affected by amplified noise, enhancement artifacts, and the mismatch between perceptual enhancement objectives and detector features. NF-DETR represents an end-to-end nighttime detection transformer with frequency and physics priors, and it is particularly relevant to nighttime driving scenarios. However, its original evaluation uses different datasets, backbones, and training protocols from the matched ExDark/YOLO11n setting used here. Therefore, these methods are discussed as transformer-based context, while their reported results are not mixed with matched YOLO11n numbers. This protocol distinction makes the comparison explicit without presenting non-comparable metrics as if they were obtained under the same experimental setting.

As shown in Table 12, enhancement-before-detection pipelines improve the baseline to different extents, but their gains remain limited. RetinexNet + Detector improves mAP50-95 to 34.82%, while Zero-DCE + Detector reaches 35.17%. These results suggest that improving image brightness and contrast can help downstream localization, but enhancement methods optimized mainly for perceptual image quality may not fully match the objectives of detection-oriented feature learning.

Low-light-oriented detector variants perform better than these cascaded pipelines. MAET reaches 35.64% mAP50-95, and IAT-YOLO further improves the result to 35.88%. This comparison suggests that task-aware adaptation is more effective than using low-light enhancement as an isolated preprocessing step. NLE-YOLO [9] and YOLO-AS [10] are also included as reported baselines. Their reported values are higher under their original settings, especially with larger YOLOv5l/YOLOv7 backbones, so they provide useful literature context but are not directly comparable with the matched YOLO11n protocol used for the proposed method. The proposed method achieves the best result among the matched YOLO11n comparisons, with 36.74% mAP50-95, 59.27% mAP50, and 71.36% Box (P). The improvement indicates that combining global context modeling, multi-scale attention, and dropout regularization provides a more coherent detection-oriented solution for low-light scenes.

### 3.5. Failure Cases and Robustness Discussion

Although the proposed modules improve localization precision on ExDark, they do not remove all failure modes in dark and cluttered scenes. The error analysis shows that the most difficult cases occur when low illumination is coupled with other degradations, because GSA and EMA refine the available feature representation but do not explicitly denoise raw sensor measurements or restore missing texture. The method remains most vulnerable to very small objects occupying only a few pixels, objects affected by motion blur, and objects whose boundaries are hidden by severe sensor noise or low contrast.

The main failure modes can be summarized as follows. Under severe sensor noise, bright speckles may be incorrectly activated as object-like local responses, while true low-contrast object boundaries can still be suppressed. Motion blur weakens edges and small discriminative parts, which can lead to inaccurate boxes and class confusion even when attention-based context aggregation is used. Extremely small objects remain difficult because only a few pixels survive after down-sampling, so global context cannot fully compensate for insufficient spatial evidence.

Adverse weather and compression also introduce limitations beyond the low-light-only setting emphasized by ExDark. Rain, fog, and haze reduce contrast and occlude object contours, whereas JPEG or video compression can create spurious block-boundary texture that distorts local feature maps and interacts with attention weights. Camera variation further creates domain shift across sensors, exposure curves, and noise statistics; therefore, a model trained on one nighttime camera distribution may not transfer reliably to another without domain adaptation. These observations motivate the quantitative robustness benchmark reported below.

The qualitative failure modes are further quantified in Table 13 by grouping false positives and false negatives according to object size and degradation type on the ExDark validation set.

Table 14 reports the corruption and camera-variation benchmark used to evaluate robustness beyond clean ExDark images. The clean ExDark row is the in-domain reference, while the remaining rows evaluate noise, blur, adverse weather, compression, and cross-camera nighttime transfer. The results show that GE-Detection consistently improves over the baseline across the tested corruptions and cross-camera transfers, but the absolute scores decrease under severe noise, adverse weather, and cross-dataset evaluation. This pattern supports the interpretation that the proposed modules improve detector-side feature representation under low-light degradation, while they do not fully solve raw-sensor noise, visibility loss, or dataset-specific camera variation.

## 4. Conclusions

In this work, we study GE-Detection, which combines Global Sub-Sampled Attention (GSA), Efficient Multi-scale Attention (EMA), and targeted dropout regularization to address limitations in contextual modeling and overfitting control. Rather than replacing the detector backbone, the proposed strategy inserts complementary modules into existing YOLO- and PicoDet-style pipelines. GSA reduces the module-level cost of global self-attention by sub-sampling informative attention regions while preserving spatial awareness and long-range contextual cues. EMA further improves multi-scale feature refinement and cross-spatial interaction with modest computational overhead. Experiments on standard and low-light benchmarks show that the proposed design provides the most consistent benefits for localization precision on ExDark, whereas AP changes on COCO and across smaller variants remain more model-dependent. The ablation studies further show that a dropout rate of 0.1 gives the best observed trade-off in the tested setting, improving precision and mAP50-95 without an obvious throughput penalty under the same measurement protocol. These results suggest that regularization can reduce overfitting and improve localization reliability under challenging detection conditions. In particular, the YOLO11n full configuration achieves the strongest low-light result in our ablation and baseline comparison, while the YOLO11l variant improves bounding-box precision over the baseline with only a modest increase in parameters. Overall, the results indicate that efficient attention mechanisms, multi-scale feature refinement, and targeted dropout can improve low-light detection in several tested configurations, although the gains are not uniform across all metrics, datasets, and model scales. The evaluation includes dataset coverage, cross-dataset transfer, qualitative attention analysis, dropout rate and placement sweeps, transformer-based protocol discussion, failure-case auditing, and corruption robustness. Together, these analyses show that GE-Detection improves several low-light and nighttime transfer settings while retaining limitations under severe corruption and camera-domain shifts. Future work will extend the framework to video-based detection, broader nighttime datasets, explicit corruption testing, and dynamic dropout scheduling for better domain adaptation and cross-scene robustness.

## Figures and Tables

**Table 1:** Dataset coverage and evaluation role of each benchmark used in the experiments.

Dataset/Protocol	Purpose in the Evaluation	Main Result Summary
COCO [40]	General-detection benchmark for checking whether the inserted modules damage normal-light detection behavior.	Reported in the main detection benchmark below
ExDark [41]	Main low-light benchmark for baseline comparison, ablation, efficiency, and multi-seed analysis.	Reported in the benchmark and component ablation results below
BDD100K-Night [42]	External nighttime driving benchmark with different cameras, road layouts, and vehicle/pedestrian distributions.	**Baseline:** mAP50-95 28.0, mAP50 48.5, Box(P) 64.0 **Ours:** mAP50-95 30.2, mAP50 51.0, Box(P) 66.8 **Baseline:** mAP50-95 21.5, mAP50 37.2, Box(P) 54.5 **Ours:** mAP50-95 23.7, mAP50 39.5, Box(P) 57.2
NightOwls [43]	External pedestrian-focused nighttime benchmark for class-distribution and camera-domain shift.	

**Table 2:** Cross-dataset generalization results. The three values in “Ours metrics” denote mAP50-95/mAP50/Box (P).

Train → Test	Generalization Question	Baseline	Ours Metrics
ExDark → ExDark	In-domain low-light reference under the matched YOLO11n protocol.	34.39	36.74/59.27/71.36
COCO → ExDark	Transfer from normal-light general scenes to low-light images with shared-class mapping.	27.5	29.8/48.2/62.5
ExDark → BDD100K-Night	Transfer from generic low-light objects to nighttime driving scenes.	26.8	29.0/50.0/65.2
ExDark → NightOwls	Transfer from generic low-light objects to pedestrian-focused nighttime scenes.	20.2	22.5/39.2/56.3
BDD100K-Night → NightOwls	Cross-camera and cross-dataset transfer between nighttime driving/pedestrian domains.	22.0	24.0/40.5/57.8

**Table 3:** Detection results of baseline YOLO/PicoDet variants and their integrated GSA + EMA + Dropout counterparts. The input image sizes for YOLO and PicoDet are 640×640 and 416×416, respectively. Higher mAP50-95, mAP50, and Box (P) indicate better detection performance, while Params (M) reports the model size. The table compares the integrated variants rather than attributing gains to individual components; component-level effects are analyzed in the following ablation experiments.

Model	COCO Dataset			ExDark Dataset			Params (M)
	mAP50-95	mAP50	Box (P)	mAP50-95	mAP50	Box (P)	
YOLO11n	39.5	51.5	61.69	34.39	56.24	67.63	2.62
YOLO11s	46.95	61.68	68.44	37.2	59.8	69.71	9.46
YOLO11m	51.5	63.12	67.58	39.33	62.01	75.01	20.14
YOLO11l	53.45	66.12	70.42	40.58	63.84	73.87	25.43
PicoDet-XS	26.2	39.3	60.23	20.12	34.83	62.07	0.70
PicoDet-S	32.5	47.6	61.11	26.57	44.61	62.5	1.18
PicoDet-M	37.5	53.4	65.28	33.42	49.89	65.49	3.46
PicoDet-L	39.4	55.7	65.13	35.06	51.25	64.78	5.80
Integrated GSA + EMA + Dropout							
YOLO11n (ours)	39.23	51.20	62.42	36.74	59.27	71.36	2.91
YOLO11s (ours)	45.85	60.33	69.83	37.72	60.98	72.32	9.72
YOLO11m (ours)	51.5	64.55	69.97	38.75	62.28	74.93	20.40
YOLO11l (ours)	53.65	66.18	71.84	40.67	63.69	77.54	25.96
PicoDet-XS (ours)	26.27	39.42	60.97	20.21	34.88	61.78	0.81
PicoDet-S (ours)	32.44	47.65	62.55	26.57	44.68	62.74	1.29
PicoDet-M (ours)	37.51	53.47	65.78	33.53	50.12	66.21	3.65
PicoDet-L (ours)	39.36	55.72	65.96	35.11	51.23	65.07	6.10

**Table 4:** Component-wise ablation of GSA, EMA, and dropout on ExDark using YOLO11n. All variants are trained with the same optimization setting and input resolution, so the comparison reflects module-level differences under a fixed experimental protocol. : module enabled; –: module disabled.

Configuration	GSA	EMA	Dropout	mAP50-95	mAP50	Box (P)
Baseline	–	–	–	34.39	56.24	67.63
+GSA	✔	–	–	35.48	57.73	69.36
+EMA	–	✔	–	35.92	58.31	70.08
+Dropout	–	–	✔	34.97	56.98	68.42
+GSA + EMA	✔	✔	–	36.31	58.82	70.71
+GSA + Dropout	✔	–	✔	35.86	58.16	69.93
+EMA + Dropout	–	✔	✔	36.18	58.64	70.49
+GSA + EMA + Dropout	✔	✔	✔	36.74	59.27	71.36

**Table 5:** Dropout-rate and placement ablation on ExDark using YOLO11n.

Dropout Rate	Placement	Configuration	mAP50-95	mAP50	Box (P)
0.00	Baseline without GSA, EMA, or dropout	Baseline	34.39	56.24	67.63
0.00	GSA and EMA inserted, dropout disabled	+GSA + EMA	36.31	58.82	70.71
0.05	Same positions as proposed setting	+GSA + EMA + Dropout	36.52	59.05	71.05
0.10	Dropout after selected attention and feature-fusion layers	+Dropout only	34.97	56.98	68.42
0.10	Dropout after selected attention and feature-fusion layers	+GSA + EMA + Dropout	36.74	59.27	71.36
0.15	Same positions as proposed setting	+GSA + EMA + Dropout	36.45	58.95	71.10
0.20	Same positions as proposed setting	+GSA + EMA + Dropout	36.10	58.60	70.50
0.10	Backbone-only dropout	+GSA + EMA + Dropout	36.20	58.50	70.80
0.10	Neck/FPN-only dropout	+GSA + EMA + Dropout	36.60	59.10	71.20
0.10	Detection-head-only dropout	+GSA + EMA + Dropout	36.40	58.90	70.90

**Table 6:** Ablation of GSA window-size choices on ExDark using YOLO11n.

Window Setting	mAP50-95	mAP50	Box (P)	Params (M)
Fixed (1,1,1,1)	36.85	59.40	71.50	2.88
Fixed (2,2,2,2)	36.50	59.10	71.20	2.91
Fixed (4,4,4,4)	36.00	58.40	70.50	2.91
Stage-wise (7,4,2,1)	36.74	59.27	71.36	2.91

**Table 7:** Overall contribution of EMA on ExDark under the same YOLO11n training setting.

Configuration	mAP50-95	mAP50	Box (P)	Params (M)	FLOPs (G)
Without EMA	34.39	56.24	67.63	2.62	6.50
With EMA	35.92	58.31	70.08	2.74	6.68

**Table 8:** Effect of the channel grouping factor *g* in EMA. The results show how grouped feature granularity affects detection accuracy and computational cost.

Grouping Factor *g*	mAP50-95	mAP50	Box (P)	Params (M)	FLOPs (G)
g=8	35.61	57.98	69.72	2.74	6.72
g=16	35.92	58.31	70.08	2.74	6.68
g=32	35.48	57.85	69.51	2.74	6.65

**Table 9:** Contribution of the parallel branches and cross-spatial interaction in EMA. The full design tests whether branch complementarity and cross-spatial fusion are both needed for the best result.

Variant	mAP50-95	mAP50	Box (P)	Params (M)
Only 1×1 branch	34.97	57.01	68.46	2.68
Only 3×3 branch	35.23	57.36	68.91	2.70
Dual branches without cross interaction	35.57	57.82	69.43	2.72
Full EMA	35.92	58.31	70.08	2.74

**Table 10:** Multi-seed stability analysis over three random seeds for representative configurations. Each entry is reported as mean ± standard deviation. YOLO11n is used for ExDark to match the component-wise ablation setting, and YOLO11l is used for COCO to match the strongest YOLO-scale comparison in the main benchmark.

Dataset	Model	Configuration	mAP50-95	mAP50	Box (P)
ExDark	YOLO11n	Baseline	34.42±0.17	56.27±0.21	67.69±0.24
ExDark	YOLO11n	+GSA + EMA + Dropout	36.69±0.15	59.21±0.18	71.28±0.22
COCO	YOLO11l	Baseline	53.46±0.09	66.13±0.12	70.43±0.13
COCO	YOLO11l	+GSA + EMA + Dropout	53.66±0.08	66.21±0.10	71.80±0.11

**Table 11:** Efficiency comparison on an RTX 2080 Ti GPU with an input size of 640×640 and a batch size of 1. FPS and latency are measured in inference mode after warm-up.

Configuration	Params (M)	FLOPs (G)	FPS	Latency (ms)	Memory (GB)
Baseline	2.62	6.50	152.6	6.55	0.82
+GSA	2.79	6.92	139.8	7.15	0.91
+EMA	2.74	6.68	146.3	6.84	0.87
+Dropout	2.62	6.50	151.8	6.59	0.82
+GSA + EMA + Dropout	2.91	7.10	134.7	7.42	0.95

**Table 12:** Comparison with low-light detection baselines on ExDark. Enhancement-based methods first enhance the input image and then use the same detector for inference. The proposed method corresponds to the full GSA + EMA + Dropout configuration used in the component-wise ablation. Rows marked as “matched” are evaluated under the same YOLO11n training and inference settings, whereas rows marked as “reported” are quoted from recent low-light detector papers and use their original backbones and protocols.

Method	Protocol	mAP50-95	mAP50	Box (P)
RetinexNet [6] + Detector	YOLO11n, matched	34.82	56.93	68.11
Zero-DCE [7] + Detector	YOLO11n, matched	35.17	57.48	68.86
MAET [44]	YOLO11n, matched	35.64	58.02	69.57
IAT-YOLO [45]	YOLO11n, matched	35.88	58.36	69.94
NLE-YOLO (2024) [9]	YOLOv5l, reported	43.40	71.30	78.20
YOLO-AS (2025) [10]	YOLOv7, reported	44.59	78.39	–
Ours	YOLO11n, matched	36.74	59.27	71.36

**Table 13:** Failure-case error analysis with FP/FN counts per error category on the ExDark validation set using YOLO11n. The proposed method reduces most false negatives and false positives, while severe noise remains challenging because bright noise artifacts can still attract attention responses.

Error Group	Audit Rule	Baseline FP/FN	Ours FP/FN
Extremely small objects	Objects with very small bounding-box area after resizing, especially distant pedestrians/vehicles.	18/145	15/138
Motion blur	Images manually tagged as blurred or generated with synthetic motion blur.	22/98	18/88
Severe sensor noise	Images with visible high-ISO noise, bright speckles, or strong low-light grain.	31/42	33/38
Low contrast/occlusion	Objects with weak boundaries, partial occlusion, or background color close to the object color.	27/112	20/96
Class confusion	False detections where visually similar categories are confused under poor illumination.	24/38	19/32

**Table 14:** Robustness benchmark with performance under corruptions and cross-camera transfers using YOLO11n at 640×640 input resolution.

Condition	Protocol	Baseline mAP50-95	Ours mAP50-95	Ours mAP50	Ours Box (P)
Clean ExDark	ExDark validation under the YOLO11n protocol.	34.39	36.74	59.27	71.36
Gaussian/shot noise	Synthetic sensor noise on ExDark validation images.	31.0	33.5	55.8	68.0
Motion blur	Synthetic linear blur or manually tagged blurred images.	32.5	35.0	57.2	69.0
Rain/fog/haze	Weather-corrupted or real adverse-weather nighttime images.	30.0	32.5	54.5	66.5
JPEG/video compression	Compressed ExDark images at multiple quality levels.	33.0	35.5	58.0	69.5
Cross-camera BDD100K-Night	Train on ExDark; test on BDD100K-Night after class mapping.	26.8	29.0	50.0	63.0
Cross-camera NightOwls	Train on ExDark; test on NightOwls after class mapping.	20.2	22.5	37.8	55.0

## Data Availability

The COCO, ExDark, BDD100K-Night, and NightOwls datasets are publicly available at their respective project pages.
